# Direct Formation of Structural Components Using a Martian Soil Simulant

**DOI:** 10.1038/s41598-017-01157-w

**Published:** 2017-04-27

**Authors:** Brian J. Chow, Tzehan Chen, Ying Zhong, Yu Qiao

**Affiliations:** 1 Department of Structural Engineering, University of California - San Diego, La Jolla, CA 92093-0085 USA; 2 Program of Materials Science and Engineering, University of California - San Diego, La Jolla, CA 92093 USA

## Abstract

Martian habitats are ideally constructed using only locally available soils; extant attempts to process structural materials on Mars, however, generally require additives or calcination. In this work we demonstrate that Martian soil simulant Mars-1a can be directly compressed at ambient into a strong solid without additives, highlighting a possible aspect of complete Martian *in*-*situ* resource utilization. Flexural strength of the compact is not only determined by the compaction pressure but also significantly influenced by the lateral boundary condition of processing loading. The compression loading can be applied either quasi-statically or through impact. Nanoparticulate iron oxide (npOx), commonly detected in Martian regolith, is identified as the bonding agent. Gas permeability of compacted samples was measured to be on the order of 10^−16^ m^2^, close to that of solid rocks. The compaction procedure is adaptive to additive manufacturing.

## Introduction

Near-future exploration to Mars connotes the technology of space construction. Permanent human settlement on Mars requires infrastructure to sustain habitats and life. A steady supply of structural materials is integral towards this effort. Of immense consequence is to examine whether such materials can be made solely through *in-situ* resource utilization (ISRU) and if so, whether energy-intensive processes such as calcination can be avoided. Previous prototyping requires energy-intensive heating^1^ or use of binders that must be shipped from the Earth^2^. Ideally, the solution should be as simple as possible.

Much of Martian regolith is formed by basaltic fines containing iron^3, 4^. The soil consists of substantial nanoparticulate iron oxides and oxyhydroxides, collectively known as npOx, responsible for its reddish hue^3–11^. Some components of npOx are too small to distinguish and are referred to as x-ray amorphous^11, 12^. A widely used Martian soil simulant, JSC Mars-1a, contains npOx^13^. A typical Mars-1a particle is composed of two phases: a basaltic body and an npOx rind^13^. The simulant serves as an analogue for Martian surface soils with high similarity in chemical composition: 45.41 wt% SiO_2_, 16.73 wt% Fe_2_O_3_ and FeO, 8.35 wt% MgO, 6.37 wt% CaO, etc. for average value of Mars regolith, versus 43.48 wt% SiO_2_, 16.08 wt% Fe_2_O_3_ and FeO, 4.22 wt% MgO, 6.05 wt% CaO, etc. for Mars-1a^14, 15^.

As with most covalently bound silicate materials, basaltic particles do not adhere to each other when compressed, unless if heated to a high temperature^16, 17^. Nanoparticulate iron oxides, however, may behave differently. Some species of npOx may develop very high specific areas upon external loadings^18, 19^. Strong bonding can be formed when large specific areas are in contact^20–22^. Based on this consideration, also motivated by the observed cohesion of Martian soils^23, 24^, we investigated whether a uniaxial load *per se* is sufficient to form solids capable of infrastructural applications.

**Figure 1:**
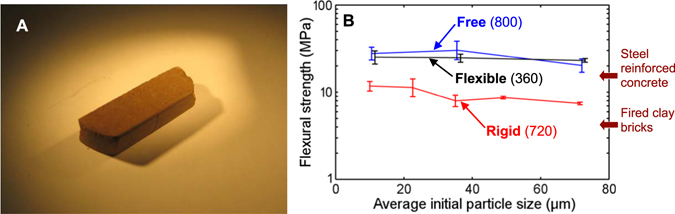
(**A**) A photo of solid Mars-1a beam quasi-statically compacted with rigid boundary condition. The curved ends show the boundaries of the steel mold. The width of the sample is ~1 cm. (**B**) Flexural strength of compacted Mars-1a as a function of the initial average particle size. The average particle sizes are computed as the nominal midpoint for each fractioned sieve size, i.e. the arithmetic mean of the bin sizes. The three series represent the three lateral boundary conditions (names in bold) with the maximum compression pressure (in MPa) inside parentheses.

Figure 1 shows the experimental results of quasi-statically compacted oven-dried Mars-1a: It is amenable to single-step rapid formation without heating or added binders. Upon a high-pressure compression, Mars-1a particles form a strong solid at ambient, with resultant flexural strengths exceeding that of typical steel-reinforced concrete or many *in situ* resource utilization (ISRU) created materials formed by adding binders^25, 26^. It is also more practical and efficient in application compared to other ISRU prototyping utilizing high temperature or laser sintering. Although Mars-1a is one of a multitude of inorganic species known to form intact solids under compression^20, 27^, its heterogeneity distinguishes npOx as a cement acting under instantaneous mechanical pressure apart from natural, long-term processes, such as fluvial concretion^18, 28^. This advance offers confidence that the self-cohesive soils seen on Mars may, in fact, be further compressed directly into high-strength structural parts.

Size fractionation and thermal treatment prepared the Mars-1a simulant prior to compaction. A reciprocating sieve separated randomly distributed simulant particles into size bins, as described in detail in the Section of Sieve Analysis in Methods; this was for determining the influence of initial particle size on the sample strength. Thermogravimetric (TGA) and CHNS/O analyses determined the water and organic contents as functions of the drying temperature, respectively (Sections of TGA, Pretreatment and Drying, and Measurement of Carbon Content in Methods; Tables S5 and S6 in Supplementary Material). Drying at 600 °C for 10 hr reduced the water content to <5 wt%. Most Martian soil, by comparison, is dry and contains <5 wt% water, although some regions exceed this^29^. The carbon content was reduced to within the null margin of error by the thermal treatment, suggesting that the influence of trace organics was trivial^30, 31^. X-ray diffraction (XRD) analysis of Mars-1a samples (Fig. S15 in Supplementary Material) suggests that the pretreatment does not induce any phase transformation or crystallinity change. A few secondary peaks disappear in the two-theta range of 20° to 30°, probably due to the loss of bonded water.

We compressed the prepared Mars-1a in a uniaxial mode between flat pistons to form solids (Section of Quasi-static Compaction in Methods). Six configurations—two rates of loading and three lateral boundary conditions around the sample—represented the compression process (Sections of Quasi-static Compaction and Impact Compaction in Methods). Loading rates were either quasi-static (with the piston velocity of a few mm/min) or impact (with the hammer velocity of a few m/s). The lateral boundary condition of compaction loading was either rigid (confined by a steel wall), free, or flexible (confined by an elastomeric wall). All boundaries were of cylindrical geometry, with the resulting compacts typically disc-shaped.

Compacted Mars-1a solids were cut into beams and subjected to three-point bending tests (Sections of Materials of Processing System, Flexural Strength Measurement, and Effects of Particle Size on Flexural Strength in Methods). The material was characterized by SEM, TEM, and XRD analyses (Sections of SEM and TEM Characterization and XRD Analysis in Methods).

We mixed neat basalt particles with Mars-1a in various proportions to determine the role that the basaltic phase plays on strength; separately, we investigated goethite and rust fines as analogues to the npOx phase^19^ (Sections of Compaction of npOx-Related Materials and Addition of Basalt Particles into Mars-1a in Methods). The testing data confirmed that the basaltic phase does not directly contribute to the sample strength, and the npOx phase indeed can behave as a binding agent.

Gas permeability of the compacted Mars-1a was measured, as described in Section of Gas-Permeability Measurement in Methods. Pressurized nitrogen flowed through a solid Mars-1a disc, and the flow rate was monitored. The Rilem-Cerembureau equation^32, 33^ was employed to calculate the permeability.

Table 1 summarizes the flexural strengths generated by each of the six processing configurations. It is remarkable that the lateral boundary condition of compaction loading significantly influences the strength and the shape of the compacted solid, specifically, the thickness-to-diameter ratio. Compared with the boundary condition, the effect of loading rate is secondary.

**Table 1:** Optimum compression pressures and the associated flexural strengths.

Boundary Condition of Compressive Loading	Quasi-static Compaction		Dynamic Compaction	
	Average Flexural Strength (MPa)	Optimum Peak Compression Pressure (MPa)	Average Flexural Strength (MPa)	Impact Pressure (MPa)
Rigid	10	720	13	~400
Free	27	800	40	>800
Flexible	25	360	50	~400

## Results

### Quasi-static Compaction and the Role of npOx

In all loading modes, the flexural strength, *R*, tends to increase with the peak compressive pressure, *P*_max_; however, when the pressure is sufficiently high, further increasing it only causes incremental improvement in *R*. The optimum peak compressive pressures associated with various boundary conditions are listed in Table 1. For self-comparison purpose, in the current study *P*_max_ is defined as the peak compressive force divided by the initial sample cross-sectional area.

Very often, strength of a compacted granular material is inversely proportional to the particle size^34^, e.g. solids formed from nanoparticles^35^. Under quasi-static loading, Mars-1a did not obey this trend: as the initial particle size changes from less than 20 μm to nearly 100 μm, *R* remains similar.

The lateral boundary condition of compression loading is a much more important factor than the initial particle size. With the rigid boundary condition, the compacted Mars-1a samples are structurally integral but the flexural strength is relatively low, comparable with ordinary clay bricks^38^. When the lateral boundary is free or flexible, *R* is nearly 3 times higher than that of rigid boundary condition. Between the free and flexible boundary conditions, the flexible boundary condition reached high *R* at much lower *P*_max_ and the resultant solid thickness was only slightly smaller than that of the rigid boundary condition, much larger than that of the free boundary condition. The strength-wise efficacy of forming inside a flexible boundary is ~150–200% greater than the free boundary for *R* ~30 MPa, taking into consideration the flexible boundary’s larger sample sizes.

**Figure 2:**
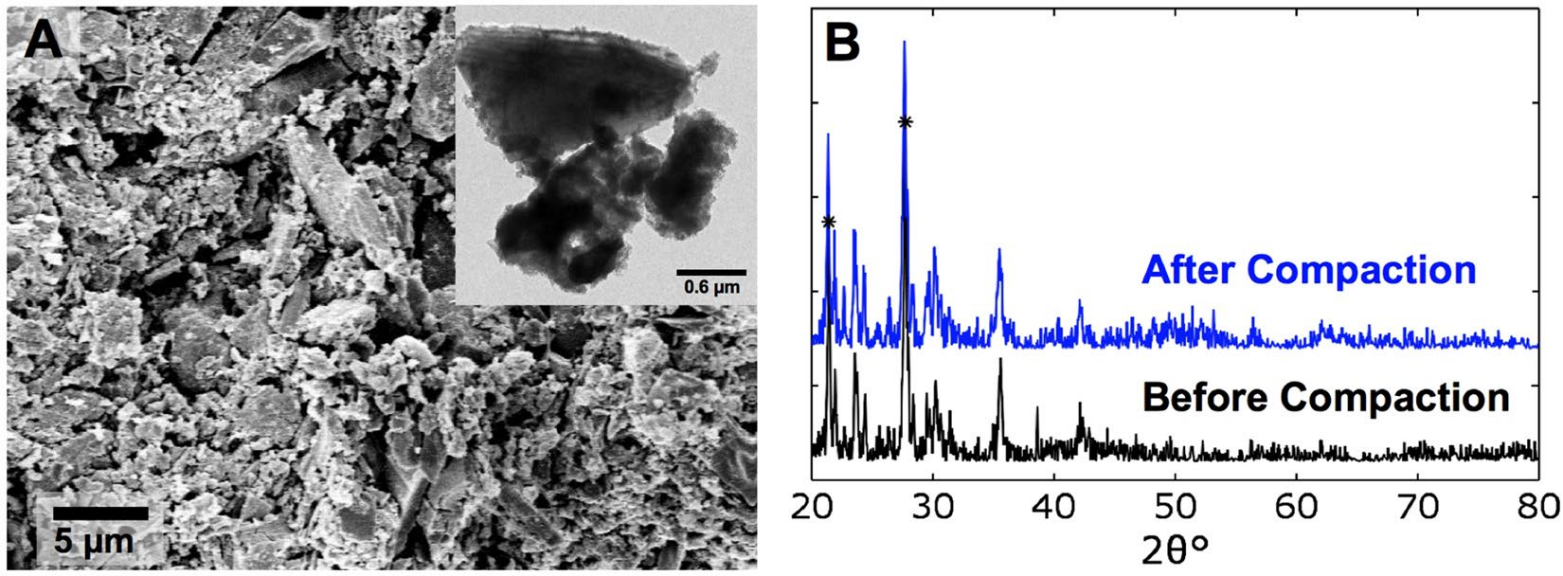
(**A**) SEM image of a solid Mars-1a fracture surface. The inset displays a TEM image of several basaltic grains bonded together; fuzzy and diffuse areas on the exterior of each particle are indicative of npOx, and the contact suggests npOx binding. The material had the initial particle size of 25–45 μm, and was compressed under 360 MPa quasi-statically with flexible boundary condition; the compression pressure was oriented horizontally in the SEM image. (**B**) Typical XRD curves of reference Mars-1a and solid Mars-1a compressed to 360 MPa with flexible boundary condition. Asterisks indicate the two main peaks of the bottom curve.

The XRD result is shown in Fig. 2. After compaction, most peaks decrease in height, including the main peaks corresponding to pyroxene minerals^39^. It may be attributed to the crushing of small crystals. The several minor peaks that grow indicate minute phase transformations of npOx into a denser form, probably goethite or magnetite^18^. Only mechanical pressure can account for these transformations, because we applied no heat during and after compression. It was reported that milling can transform various FeOOH species into hematite^18, 40^.

Quasi-statically compacted Mars-1a lost strength when diluted with basalt (Table S3 in Supplementary Material). As the amount of basalt additive exceeds 75 wt%, the compacted material disintegrates spontaneously and R is essentially zero. When goethite particles are compressed with rigid boundary condition, the samples show similar flexural strengths to Mars-1a (Table S2 in Supplementary Material).

### Impact Compaction

**Figure 3:**
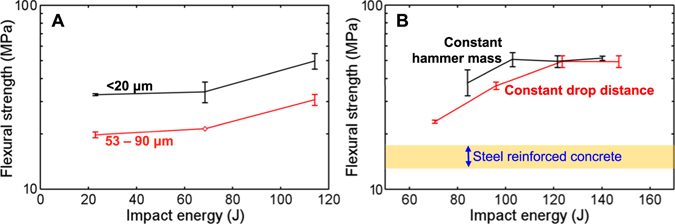
(**A**) Flexural strength of compacted Mars-1a as a function of the impact energy; the lateral boundary is free during the impact. The two series are labeled with their initial particle sizes; they obey similar trends, with the smaller particle size resulting in a higher strength. Impact energy was varied by adjusting the drop distance. (**B**) The flexural strength of compacted Mars-1a as a function of the impact energy; the lateral boundary is flexible during the impact; the initial particle size is 25–45 μm. The two series are distinguished apart by either controlling the hammer mass or the drop distance to adjust the impact energy. Typical range of flexural strength of steel-reinforced concrete is shown for comparison^37^.

Upon impact compaction, the free or flexible boundary condition again yields high *R*. As shown in Table 1 and Fig. 3, the free boundary condition produced the strongest samples, albeit with higher peak pressure than the flexible boundary condition. When the impact pressure is comparable with the quasi-static pressure, the resultant flexural strength of dynamically formed Mars-1a specimens is higher than those which were quasi-statically formed, implying that the bonding formation process is faster than the characteristic time of impact compaction, around a few milliseconds. Figure 3(A) shows that as the initial particle size, *D*, decreases from the range of 53–90 μm to 25–45 μm, *R* significantly increases. This increase differed from the observed *R*-*D* relation when the loading was quasi-static.

Figure 3(B) shows that *R* is determined by the impact energy with other parameters unchanged, no matter whether the impact energy is adjusted by the hammer mass or the impact velocity.

Figure S9 in Supplementary Material shows that the peak stress in the impact pulse is about ~400 MPa for a 120-J drop. It led to a flexural strength ~50 MPa (Fig. 3(B)), significantly greater than *R* ~30 MPa in the quasi-static case for a similar *P*_*max*_.

### Nitrogen Permeability

The nitrogen permeability of compacted Mars-1a is on the order of ~10^−16^ m^2^ (Table S4 in Supplementary Material). It suggests that the compacted material is somewhat similar to dense rocks^41, 42^, and can be adapted to an atmospheric habitat.

## Discussion

The phenomenon that the flexural strength of quasi-statically compacted Mars-1a, *R*, is insensitive to the initial particle size, *D*, may be associated with the high compressive pressure, *P*_max_. The crushing pressure of basalt grains is typically 1–10 MPa^36^, much below *P*_max_. Hence, Mars-1a particles are crushed into small pieces. For instance, as *D* is 25–45 μm, most of the particles are smaller than 10 μm after compaction, as shown in Fig. 2(A) and Fig. S16 in Supplementary Material. Consequently, the properties of compacted solid are unrelated to the initial particle size distribution.

An even dispersion supports the notion of continuous transport of npOx around voids, given that crushing of particles occurs throughout compression. Added basalt essentially served an inert filler material; without sufficient npOx, it cannot be responsible for the strength. The result affirms that npOx binds Mars-1a. Decreasing strength with added basalt also rules out the hypothesis of mechanical interlocking of particles^21^. Goethite is able to achieve strong bonding by virtue of the relatively high specific area, making it a plausible constituent of npOx.

With the flexible boundary condition, the particle motion is localized. Clearly, during compaction it is critical to allow rotation and transverse motion of Mars-1a particles, so as to maximize the effective contact area and to promote bond formation. Excessive particle motion, however, may introduce defects, which explains why the free boundary compensates with higher pressure to achieve similar *R* as the flexible boundary.

For impact formation, the particle size dependency of flexural strength may be related to the effects of inertia and npOx availability. Larger particles fall into less-than-optimal arrangements, because rotational inertia depends strongly on the size of particles. Larger particles also have a larger solid volume-to-area ratio, so that less npOx is available to bond simulant particles. The durations of quasi-static and dynamic loadings are separated by about 5 orders of magnitude^38^.

To explain the permeability of the material, we propose that flow channels of nitrogen are formed by microscopic defects among particles, which, as a first-order approximation, may be modeled as cylindrical tubes carrying Poiseuille flow. Dividing the cross-sectional area of the solid, *A*_*s*_, by the average area of a single particle, *A*_*p*_, we assume that there are ~10^5^ particles over the cross-section. In a face center cubic structure, each particle contributes 1 defect. The radius of each flow channel is *r* ~10 μm, assuming defect sizes on the order of particle size. Poiseuille’s equation for channel flow can be combined with the Rilem equation to give $k=\frac{\pi }{4}(\frac{{r}^{4}}{{A}_{s}})(\frac{{P}_{o}}{{P}_{i}+{P}_{o}})$, where *P*_*o*_ is ambient pressure and *P*_*i*_ is the upstream pressure. The result is ~10^−17^ m^2^, which is in reasonable agreement with the experimental data.

In summary, in ambient environment, Mars-1a Martian soil simulant can develop considerable strength around 30–50 MPa upon one-step high-pressure compaction, with npOx being the bonding agent. The compaction can be performed either quasi-statically or through impact. The processing procedure may be generalized; e.g. the compacted solid can be further strengthened by reinforcements. Although compression-forming represents a novel process for npOx, the process itself may be adapted to a number of other Martian and terrestrial minerals. Such minerals include clays and evaporite salts. Both quasi-static and impact compaction processes are compatible with additive manufacturing, which will be a focus of future study; it is critical to the production of large-sized structural parts, wherein the material may be added incrementally.

## Methods

### Sieve Procedure

Using a drying oven (VWR-1330GM), we subjected 500-ml batches of Mars-1a simulant (Orbitec JSC Mars-1a, <1 mm) to a temperature of 105 °C for 24 hr (Fig. S1 in Supplementary Material). Then, a mechanical sieve (Tyler Rotap RX-29, 350 W) separated the simulant into bin sizes using 90 μm, 53 μm, 45 μm, 25 μm, and 20 μm meshes. The average particle size, *D*, refers to the arithmetic mean of the bin sizes.

### Thermogravimetric Analysis (TGA)

A thermogravimetric analyzer (Perkin Elmer Pyris 1 TGA) heated and weighed 15 mg of nitrogen-purged simulant, using a ramp rate of 5 °C/min to a maximum temperature of 1000 °C held for 20 min. The sample was subsequently furnace-cooled to ambient temperature. Three measurements were averaged and the result is shown in Fig. S2 in Supplementary Material.

### Pretreatment and Drying

Mars-1a simulants were transferred in crucibles, protected with aluminum foils, and subjected to 600 °C for 12 hr inside a furnace (Carbolite CTF 12/75/700), followed by furnace-cooling to ambient temperature.

### Quasi-Static Compaction

#### Rigid boundary condition

Mars-1a simulants were transferred inside the chamber of a cylindrical steel mold fitted with a bottom piston. Pistons were either 19.1 mm or 12.7 mm in diameter, with the latter used for higher pressures. The top piston was inserted slowly on top of the simulant, covering the mold (Fig. S3 in Supplementary Material). A uniaxial testing machine (Instron 5582) loaded the assembly to the maximum compression pressure (*P*_max_) at a rate of 6 mm/min. The compression pressure was calculated as the compressive force divided by the cross-sectional area of the piston. The peak pressure lasted 5–10 s, and relieved <1 sec. A 1-ton arbor press extracted the solid Mars-1a from the assembly.

#### Free boundary condition

Compression of Mars-1a simulants without a lateral confinement was achieved by pistons acting on undersized precursors. For the purposes of our geometry, these precursors were disc-shaped pellets with the height of 2–3 mm. Precursors were made by pressing simulant in relatively undersized dies (8.7 or 19.1-mm piston diameter) to 100 MPa. We then reinserted precursors concentrically into larger dies between matching pistons (19.1 or 50.4-mm-diameter, respectively) and loaded the assemblies with the uniaxial testing machine (Instron 5582 or SATEC M600XWHVL, respectively) at 6 mm/min to *P*_max_, as depicted in Fig. S4 in Supplementary Material. The compacted sample was then manually removed.

#### Flexible boundary condition

Mars-1a simulant was transferred inside a flexible tube (Finger Lakes Extrusion Clearflex 70–1 8170–2590) of height 30 mm, fitted on the bottom with a steel piston (19.1 mm diameter) of matching size. The piston translated under force when engaged with the tube, but did not slip; the flexible tube had the outer diameter of 19.1 mm and the thickness of 3.2 mm. The second piston was placed on top of the simulant, and the assembly was loaded to *P*_max_ using the uniaxial testing machine (Instron 5582) at 6 mm/min (Fig. S5 in Supplementary Material). No hydrostatic control was applied to the exterior of the tube. Typical load-displacement curves are shown in Fig. S6 in Supplementary Material.

### Impact Compaction

#### Free boundary condition

We generated precursors of thickness 2–3 mm using a steel mold with 8.7 mm piston diameter. Precursors were transferred inside a 19.1-mm-diameter steel mold press-fitted on the outside with a polyurethane jacket (Pleiger Plastics MCTB9595145B), positioned concentrically between matching pistons. The steel mold’s top face was 1 mm lower than that of jacket’s when their bottom faces were flush. To protect hardware, a cushion layer, made of 1 g aluminum foil pressed inside a separate 19.1-mm-diameter mold, was placed above the top piston and another below the bottom piston. The jacketed assembly was then secured inside a fixture, vertically collinear with a guide tube (Fig. S7 in Supplementary Material). The guide tube had dimensions of 76.2 mm outer diameter and 4.8 mm wall thickness. The guide tube lengths were 1.5 m, 0.9 m, or 0.3 m in separate trials, and the longest length was stabilized vertically by securing to the ceiling. A 7.6-kg steel impact hammer fell through the guide tube, striking the assembly on the cushion layer. No simulants escaped the mold assembly during impact. We calculated the impact energy as *V* = *mgh*, where *V* is the gravitational potential energy, *m* is the mass of the hammer, *g* is the standard gravity, and *h* is the drop distance.

#### Flexible boundary condition

Mars-1a simulant was inserted inside a flexible tube (Saint Gobain Tygon R-3603 AAC00037) and capped on opposite ends with 12.7-mm-diameter steel pistons. The tube was 12 mm in diameter, 24 mm in wall thickness, and 25.4 mm in height. The piston did not slip when engaged with the tube. The total initial height of the simulant inside the tube was ~25 mm. The capped tube was outfitted with an elastomer jacket (Thomas Scientific 9544T65). The jacket had dimensions of 38 mm outer diameter, 19 mm inner diameter, and 74 mm height. The tube containing simulant was pushed down the jacket such that the bottom piston was flush with the jacket. The bottom of the jacketed assembly was then positioned inside a steel cap, of dimensions 38.5 mm inner diameter, 10.4 mm bore depth, and 25.4 mm thickness. To protect the hardware, we formed ~1 g aluminum foil into a 12.7-mm-wide, 2-mm-thick cushion layer at 40 MPa with another die and placed it above the top piston. The entire assembly with the simulant, tube, pistons, jacket, cap, and cushion is shown in Fig. S8 in Supplementary Material. We transferred the assembly inside the chamber of a drop tower (Instron CEAST 9350), with the hammer mass from 2.8 kg to 5.8 kg and the drop distance from 1.8 m to 3.0 m. The hammer mass included the weight of the tup frame, transducer, hammer head, and detachable weights. No simulants escaped from the flexible tube during impact. The compacted sample was harvested by isolating it from surrounding loose material. Figure S9 in Supplementary Material shows a typical impact pulse.

### Compaction Condition and Devices

Compaction and testing were performed at ambient; samples remaining overnight were stored at elevated drying temperature of 105 °C. Steel molds used for compression with the rigid boundary condition were of hardness R_C_ 50. Pistons attained a hardness of R_C_ 60, and end faces were regularly polished with 13-μm sandpapers. Clearance between piston and mold amounted to 0.05 mm or less.

### Flexural Strength Measurement

Compressed solids were cut and reduced with 26-μm and 13-μm abrasives to beams with dimensions described by Table S4 (Fig. S10 in Supplementary Material). Prior to testing, we lightly chamfered the lengthwise edges using 13-μm abrasive to guard against edge defects.

A fixture held the beam by its ends to represent a simply-supported condition. Distances between the supports were 5.0, 9.8, or 15.2 mm depending on the size of the beam. A single top fixture applied a point load at midspan in all cases. Testing was performed with a 2-kN load cell of 0.2% full-scale sensitivity, on a Type-5582 Instron machine. The flexural test loaded the beam at a rate of 6 mm/min. Figure S11 in Supplementary Material shows a typical load-displacement curve. The flexural strength, *R*, was calculated as^17^: $R=\frac{3{F}_{m}L}{2b{t}^{2}}$, where *F*_m_ is the peak loading, *L* is the distance between supports, *b* is the beam width, and *t* is the beam depth. Four valid tests constituted the minimum to calculate a standard deviation.

The total time between the heat treatment and the completion of the flexural strength test did not exceed 12 hours. While samples idled, they were maintained inside a sealed container in dry air at 110 °C.

### Effects of Particle Size on Flexural Strength

We sieved, dried, and compressed Mars-1a simulant following the procedure described in Sections of Sieve Analysis, Pretreatment and Drying, and Quasi-Static Compaction (Rigid boundary condition) in Methods. Two data series were generated: one compressed at 360 MPa and the other at 720 MPa. We procedurally discarded the high datum and low datum, and at least four data points generated the standard deviations. The two series were combined into one data set by using error propagation^43^. The results are plotted in Fig. S12 in Supplementary Material.

### Compaction of npOx-Related Materials

Rust fines were prepared using steel shavings exposed to aqueous solution of 0.2 M acetic acid in a glass dish exposed under ambient condition for 168 hr. The rust fines were separated from the metal by agitation in a 200 ml beaker filled with ethanol, and then silted for 60 sec. The remaining supernatant fluid was transferred into a second beaker, and silted again for 24 hrs. Once isolated, the fines were further rinsed with distilled water and acetone repeatedly. Oven-drying was performed at 80 °C, 350 °C, or 500 °C overnight prior to compression. Particle sizes of freshly generated rusts was ~30 nm^19^, bypassing the need for mechanical sieving.

Goethite (Sigma-Aldrich 71063–100 G) was used directly as-supplied. The goethite was not dried prior to compaction, due to the sensitivity of transformation temperature. Like the rust fines, goethite — itself a constituent of rust^18^— had a relatively small crystallite size and was not sieved.

Both materials were quasi-statically compacted using the rigid boundary condition and tested as described in Section of Flexural Strength Measurement in Methods. The testing results are shown in Table S2 in Supplementary Material.

### Addition of Basalt Particles into Mars-1a

Basalt rock (Washougal Quarry) was cleaned with deionized water, dried at 105 °C for 2 hr, and comminuted with a mortar and pestle. The basalt particles were then sieved and dried following the procedure described in Sections of Sieve Analysis and Pretreatment and Drying in Methods. The 25–45 μm size fraction was mixed with the same size fraction of Mars-1 simulant, which had been sieved and dried. The prepared mixture was compressed and tested following the procedure described in Sections of Quasi-Static Compaction (Rigid boundary condition) and Flexural Strength Measurement in Methods. The compositions were 10 wt%, 25 wt%, 50 wt%, or 75 wt% basalt particles, balanced by Mars-1a. The results are summarized in Table S3 in Supplementary Material.

### Gas-Permeability Measurement

Compressed Mars-1a solids were generated according to the procedure described in Section of Quasi-Static Compaction (Flexible boundary condition) in Methods using the 19.1-mm-diameter mold. The resultant solid was ground into a round disc, with the final pass using 13-μm abrasive. In the final form, the discs were 12.7 mm in diameter and ~3 mm in thickness.

The permeability test stand consisted of a flexible clear tubing connected to a 138-kPa gauge nitrogen gas source. A break in the tubing allowed the disc sample to be inserted and resealed with hose clamps. Vacuum grease (Dow Corning) and a cylindrical piston aided in inserting the disc. The end of the tube led to an upside-down 100-ml graduated cylinder full of water. Leaks were precluding by performing controls with a reference steel piston; no bubbles were observed in the graduated cylinder for 30 min. Permeability was calculated as^32^: $k=Q(\frac{2\mu t}{A})(\frac{{P}_{o}}{{P}_{i}^{2}-{P}_{o}^{2}})$, where *μ* is the dynamic viscosity of nitrogen at 0.0000176 Pa-s, *t* is the sample thickness, *A* is the circular cross-sectional area, *P*_*o*_ is ambient atmospheric pressure (~101.3 kPa), and *P*_*i*_ is the nitrogen source absolute pressure (~239.3 kPa). Table S4 in Supplementary Material lists the measurement results and the calculated permeability for each sample.

### Measurement of Carbon Content

Mars-1a simulant was separately dried at 350 °C and 500 °C for 12 h, and subjected to CHNS/O analysis by using a Perkin Elmer PE2400-Series II Analyzer to detect the remaining carbon content. The results are shown in Table S5 in Supplementary Material, together with the carbon content of as-received Mars-1a.

Flexural strength tests were conducted on compacted samples produced through the procedure described in Section of Quasi-Static Compaction (Rigid boundary condition) in Methods, using Mars-1a particles dried at either 230 °C or 500 °C. The oven-drying procedure followed Section of Pretreatment and Drying in Methods. The decrease in remaining carbon content with increasing temperature is shown in Table S5 in Supplementary Material.

### SEM and TEM Characterization

Fracture surfaces of tested Mars-1a samples were observed in a FEI XL30 Scanning Electron Microscope (SEM). Figure 2(A) in the main text and Figs S13 and S14 in Supplementary Material show typical SEM images.

About 20 mg of powders were harvested from the fracture surface of a Mars-1a sample compacted quasi-statically with flexible boundary condition. They were observed under a Hitachi HD-2000 (TEM). The inset in Fig. 2(A) shows a typical TEM image.

### XRD Analysis

X-ray diffraction (XRD) analysis was performed on as-received Mars-1a powders, dried Mars-1a powders, and Mars-1a powders harvested from compacted samples (all about 20 mg) using a Rigaku Miniflex-II XRD machine. Typical results are shown in Fig. 2(B) in the main text and Fig. S15 in Supplementary Material.

## Electronic supplementary material

Supplementary Information
