# Supplementary material for: Direct Formation of Structural Components Using a Martian Soil Simulant
Source: Sci Rep. 2017 Apr 27;7:1151. doi: 10.1038/s41598-017-01157-w (PMC5430746; doi:10.1038/s41598-017-01157-w)
Supplement: Supplementary file 1 — Supplementary Information [file 41598_2017_1157_MOESM1_ESM.pdf]

Supplementary Material

for

Direct Formation of Structural Components Using a Martian Soil Simulant

Brian J. Chow,[1] Tzehan Chen,[2] Ying Zhong,[2] Yu Qiao[1,2,*]

[1] *Department of Structural Engineering, University of California – San Diego, La Jolla, CA 92093-0085, U.S.A.*
[2] *Program of Materials Science and Engineering, University of California – San Diego, La Jolla, CA 92093, U.S.A.*

* Corresponding author. E-mail: yqiao@ucsd.edu

This PDF file includes:

Figures S1 to S16
Tables S1 to S6

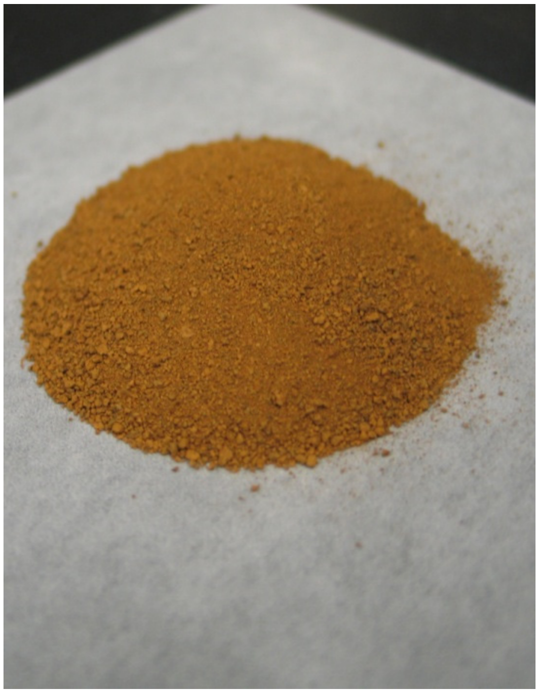

Fig. S1 An 8-cm-wide pile of oven-dried Mars-1a simulant.

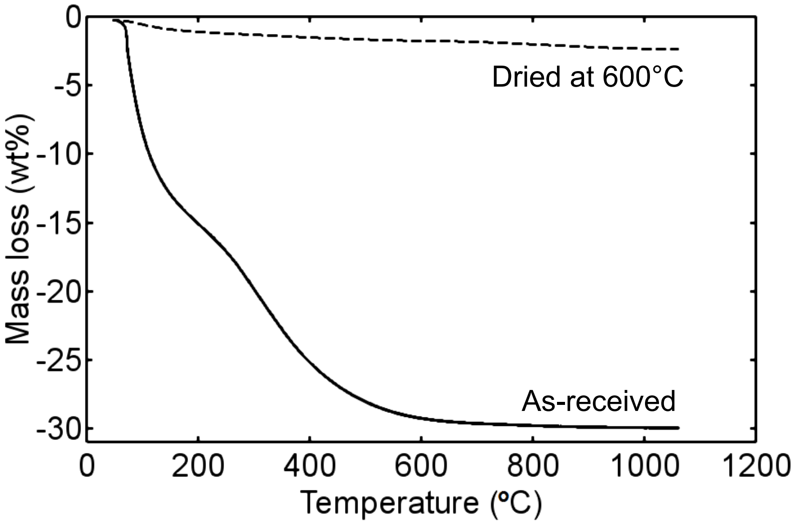

Fig. S2 Typical TGA curves of as-received and dried Mars-1a samples.

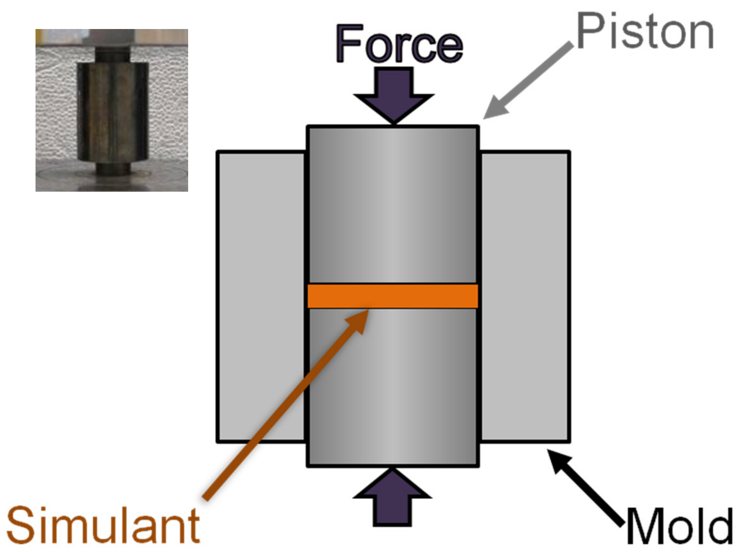

Fig. S3 A schematic of the compression setup with rigid boundary condition; the inset at the upper-left corner is a photo of the installed assembly with 19.1-mm-diameter pistons.

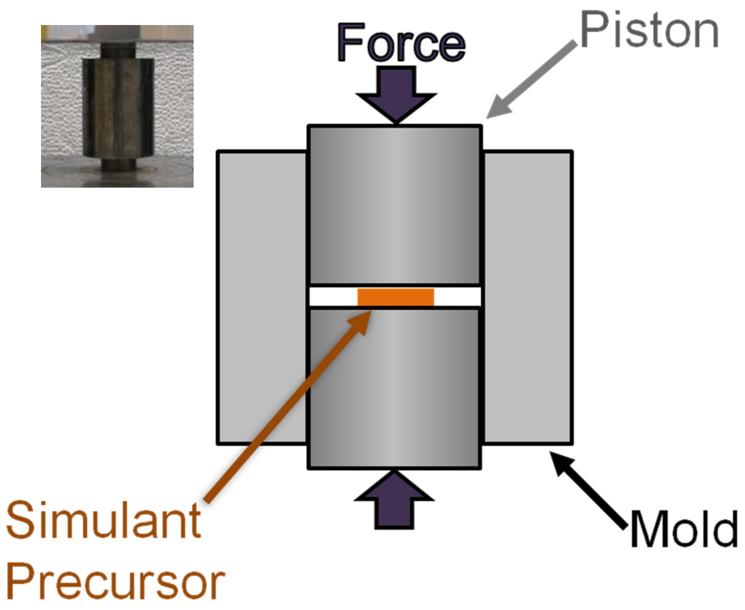

Fig. S4 A schematic of the compression setup with free boundary condition; the inset at the upper-left corner shows the installed assembly with 19.1-mm-diameter pistons.

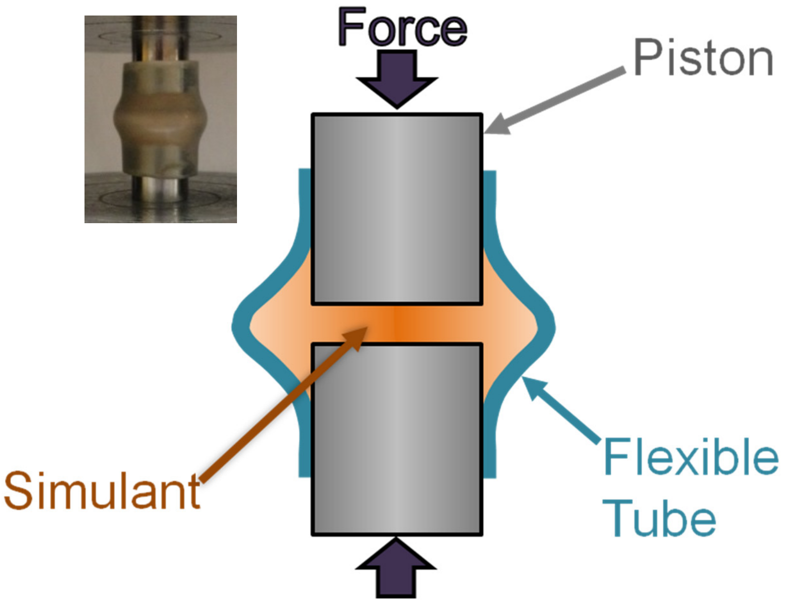

Fig. S5 A schematic of the compression setup with flexible boundary condition; the inset at the upper-left corner shows the installed assembly with 19.1-mm-diameter pistons.

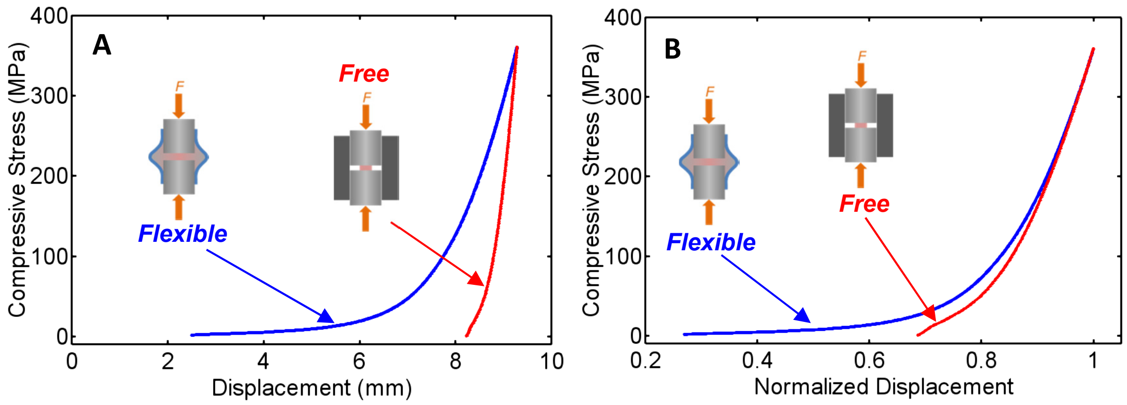

Fig. S6 Typical load-displacement curves using the free boundary and flexible boundary conditions: (A) absolute displacements and (B) normalized displacements. Normalization of the data in (B) was performed in four steps. First, the data for low displacements and low stresses close to zero were truncated. Second, the compressive displacements were normalized by the final displacement. Third, the abscissa for the free boundary condition was rescaled to match the right-hand side linear elastic slope of the flexible boundary condition, to correct for geometric stiffness differences between their area-to-thickness ratios. Lastly, the right-hand extrema were brought to coincide with one another.

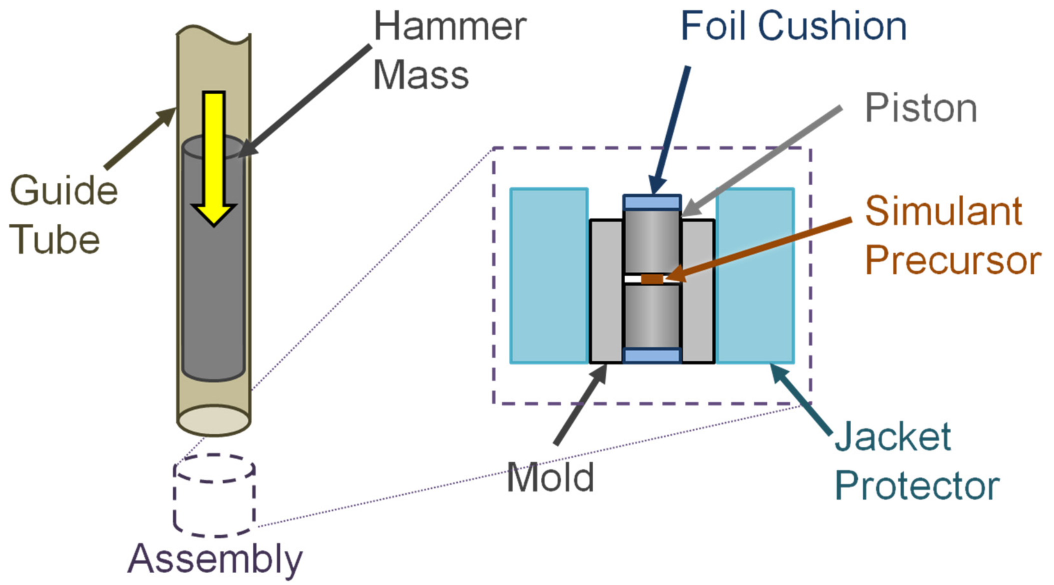

Fig. S7 Schematic of the dynamic compaction setup with free boundary condition. On the left, a hammer mass falls onto an assembly, assisted by the guide tube. The bottom of the guide tube and the assembly are joined by a rigid fixture (not shown) to align the impact. The assembly is composed of the components labeled by the dashed box on the right; an interference fit secures the mold to the jacket.

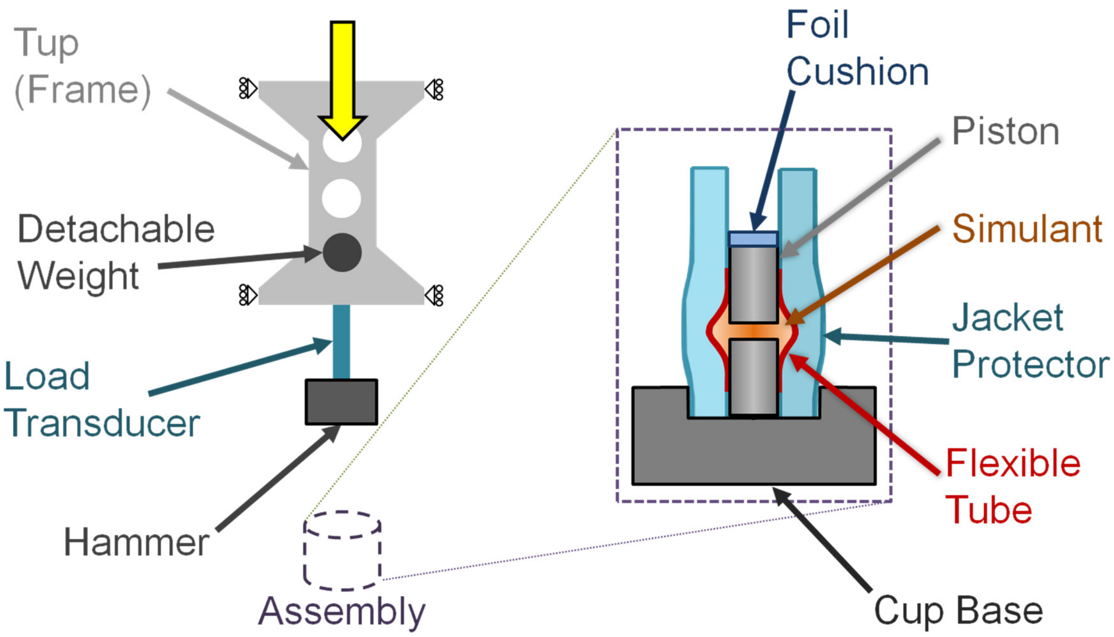

Fig. S8 Schematic of the dynamic compaction setup with flexible boundary condition. The tup, weight, transducer, and hammer fall vertically to strike the assembly, as shown on the left. The assembly components are shown on the right. We introduced interference between the flexible tube and jacket by lightly preloading the pistons, producing a bulge near the base. The foil cushion is added on top after the preload.

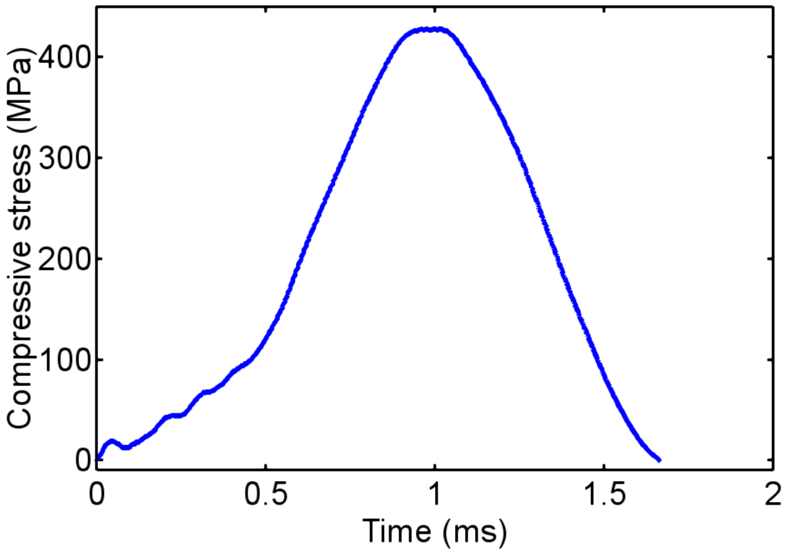

Fig. S9 A typical 120-J impact pulse, measured by the CEAST 9350 load transducer. The typical pulse duration is on the scale of microsecond (ms).

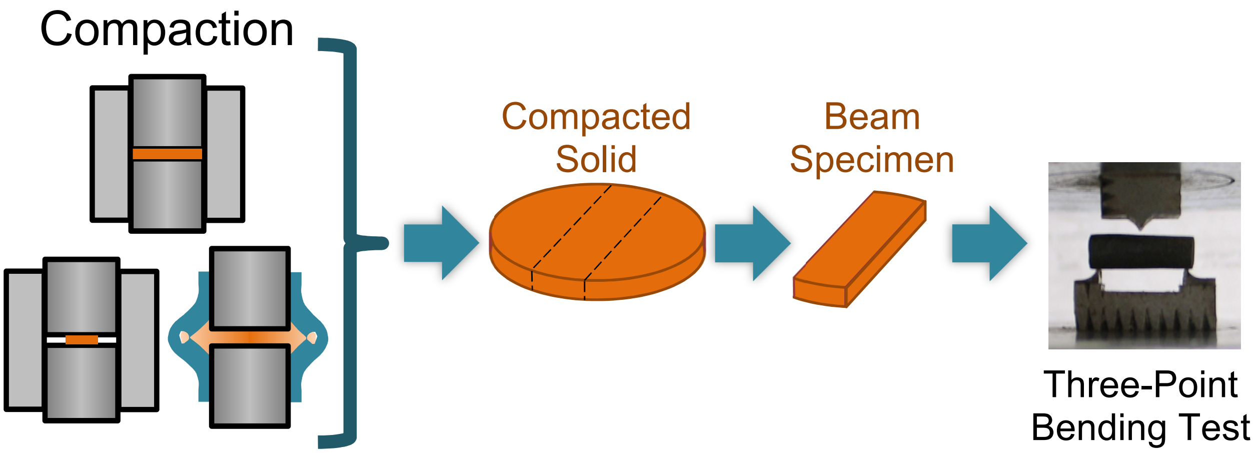

Fig. S10 Beam sample preparation process. Left side depicts the three apparatuses, each representing a different boundary condition during compression. Middle-left step depicts a solid disc after removal from the compression apparatus; dashed lines represent where the material is removed. Middle-right step depicts a beam specimen (Table S4 lists the typical dimensions of the beam specimens). Right side is a photograph of the 3-point bending test setup, using a fixture with the support span of 15.2 mm, as positioned between the testing machine platens.

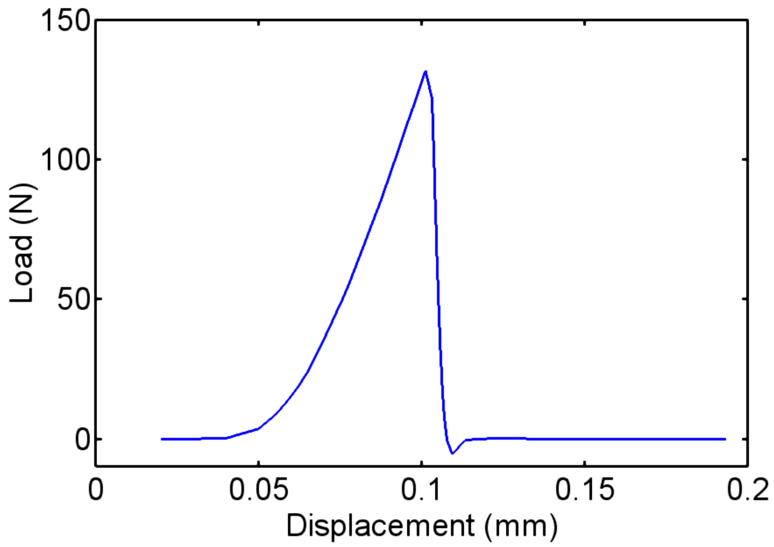

Fig. S11 A typical load-displacement curve of 3-point bending test. The loading develops a linear-elastic ramp to a maximum peak where failure occurs; the maximum peak is the force used to compute the flexural strength, *R*.

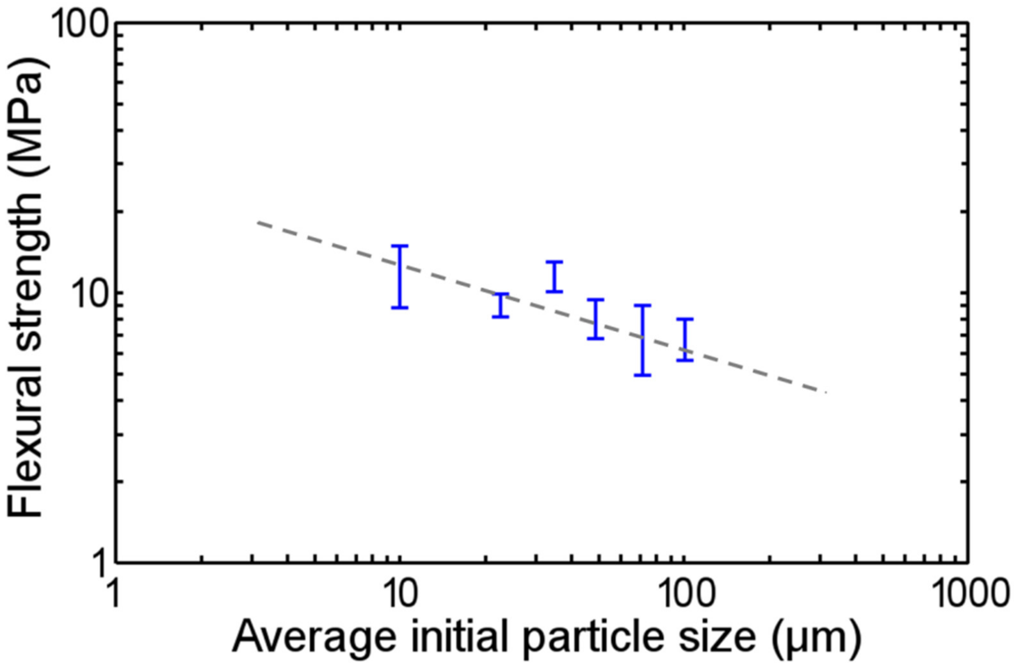

Fig. S12 Flexural strength of Mars-1a samples compacted with rigid boundary condition, as a function of the initial average particle size. The least-squares regression result is shown by the dashed line.

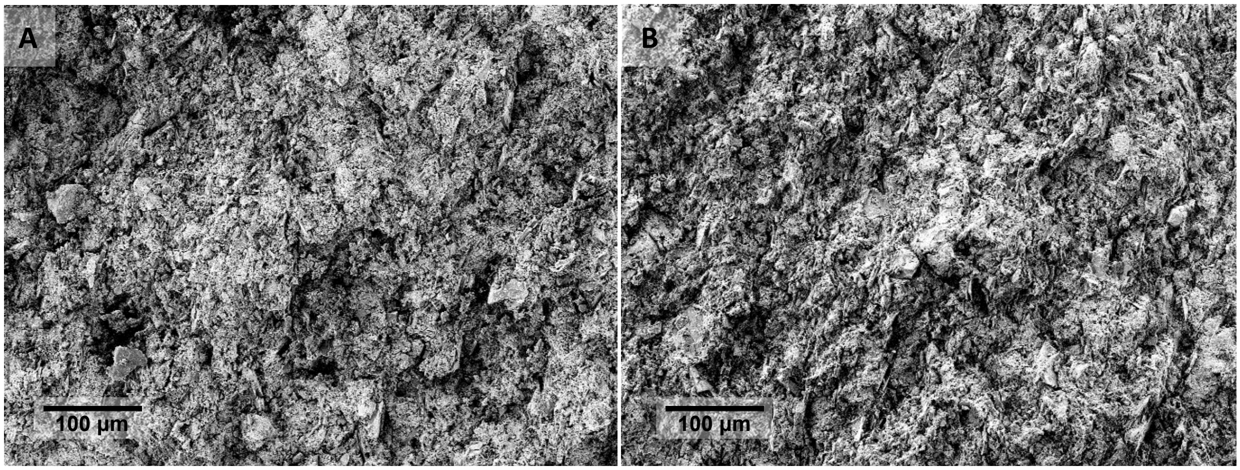

Fig. S13 SEM images of fracture surfaces at two different locations (A and B) of a Mars-1a sample compacted with flexible boundary condition. The initial particle size is 25-45 µm.

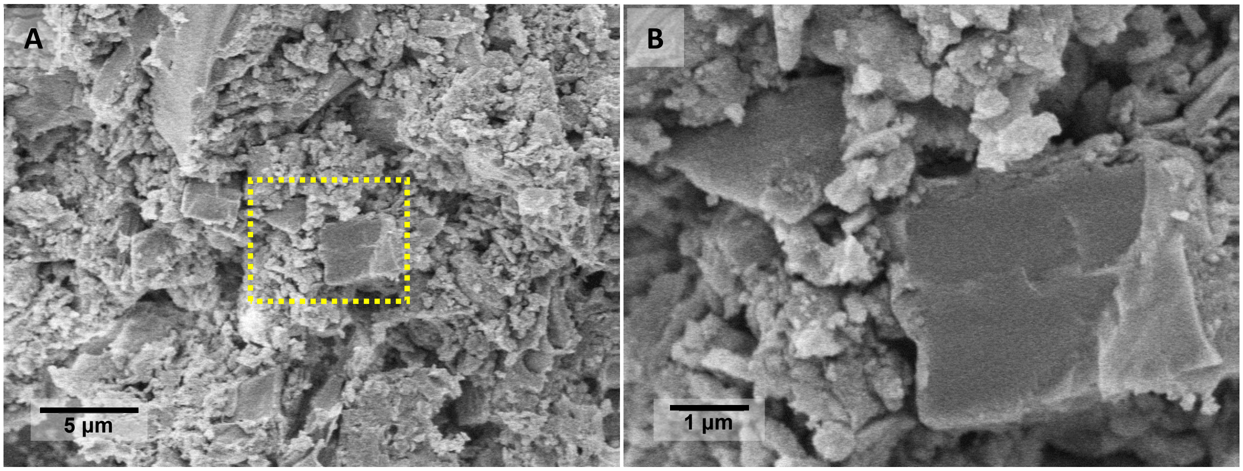

Fig. S14 (A) SEM image of a fracture surface of Mars-1a compacted with flexible boundary condition. (B) The magnified view of the framed area in (A).

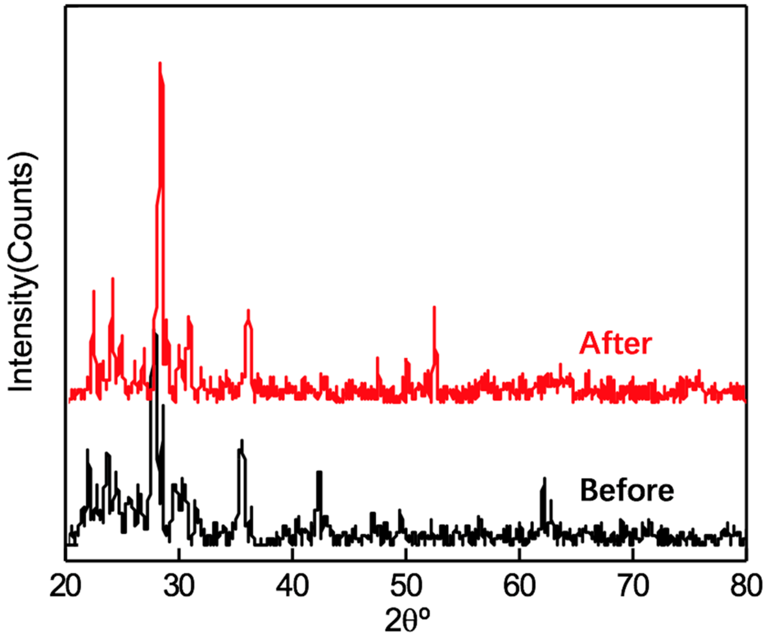

Fig. S15 XRD results of Mars-1a samples before and after the preheating treatment at 600 ^{o}C for 10 h.

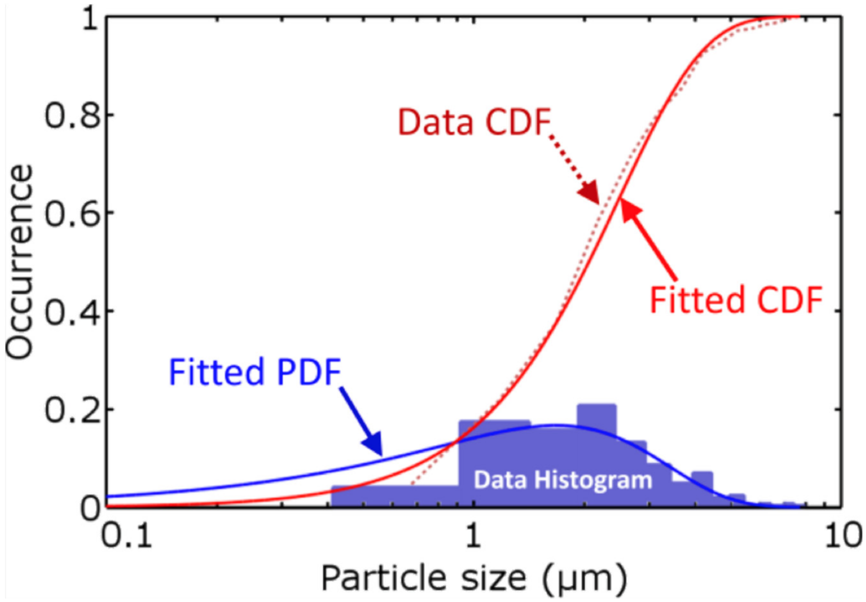

Fig. S16 Weibull Fitting and distribution curves represent crushed particles of Mars-1a compressed at 360 MPa. The initial particle size was 25-45 μm. Distributions of the particle diameters are generated by taking the square roots of the areas calculated from SEM images. A probability density function (PDF) describes the frequency of occurrence per range of particle size. Integrating the PDF gives the cumulative density function (CDF), which describes the weight fraction of all particles smaller than a particular size.

Table S1 Typical beam specimen sizes

Boundary Condition of compaction loading	Length (mm)	Width (mm)	Thickness (mm)
Rigid	19	5	2
Free	5	2	0.6
Flexible	10	4	3

Table S2 Flexural strengths of compacted iron oxides

Sample No.	Material	Drying Temperature (°C)	Flexural Strength (MPa)
1	Rust Fines	80	7.46
2	Rust Fines	80	8.78
3	Rust Fines	80	7.29
4	Rust Fines	80	9.85
5	Rust Fines	80	7.32
6	Rust Fines	350	5.44
7	Rust Fines	500	6.27
8	Rust Fines	500	5.06
9	Rust Fines	500	4.96
10	Rust Fines	500	4.87
11	Rust Fines	500	6.06
12	Rust Fines	500	6.41
13	Goethite	25	12.40
14	Goethite	25	13.41

Table S3 Flexural strengths of compacted mixtures of Mars-1a and basalt particles

Sample No.	Basalt Content (wt%)	Flexural Strength (MPa)
M1	0	8.43
1	10	6.25
2	10	6.45
3	25	8.94
4	25	6.51
5	50	2.38
6	50	3.06
7	75	2.33
8	75	1.32
BA	100	0

Table S4 Permeability test results

Sample No.	Thickness (mm)	Cross-sectional Area (mm^2)	Elapsed Time (s)	Volume (ml)	Permeability ($\times 10^{-16}$ m^2)
1	2.88	144	908	101	1.70
2	2.61	141	785	85	1.52
3	2.70	138	737	86	1.73
4	2.62	140	971	120	1.76

Table S5 Remaining carbon contents of Mars-1a after drying at various temperatures

Drying Temperature (°C)	No. of Specimens	Remaining Carbon Content (wt%)
22	3	3.16
350	3	0.68
500	3	0.30

Note: The resolution of measurement is 0.3 wt%.

Table S6 Flexural strengths of compacted Mars-1a dried at 230 ^oC or 500 ^oC

Drying Temperature (°C)	No. of Specimens	Mean Flexural Strength (MPa)	Standard Deviation (MPa)
230	8	7.79	0.79
500	4	7.72	0.72
